# Short-term Effects of Risperidone Monotherapy on Spontaneous Brain Activity in First-episode Treatment-naïve Schizophrenia Patients: A Longitudinal fMRI Study

**DOI:** 10.1038/srep34287

**Published:** 2016-10-04

**Authors:** Mao-Lin Hu, Xiao-Fen Zong, Jun-Jie Zheng, Spiro P. Pantazatos, Jeffrey M. Miller, Zong-Chang Li, Yan-Hui Liao, Ying He, Jun Zhou, De-En Sang, Hong-Zeng Zhao, Lu-Xian Lv, Jin-Song Tang, J. John Mann, Xiao-Gang Chen

**Affiliations:** 1 Department of Psychiatry, the Second Xiangya Hospital, Central South University, Changsha, Hunan, China; 2 Division of Molecular Imaging and Neuropathology, New York State Psychiatric Institute and Departments of Psychiatry and Radiology, Columbia University, New York, NY, USA; 3 Mental Health Institute of the Second Xiangya Hospital, Central South University, Chinese National Clinical Research Center on Mental Disorders (xiangya), Chinese National Technology Institute on Mental Disorders, Hunan Key Laboratory of Psychiatry and Mental Health, Changsha, Hunan, China; 4 Key Laboratory for NeuroInformation of the Ministry of Education, School of Life Science and Technology, University of Electronic Science and Technology of China, Chengdu, China; 5 Division of Molecular Imaging and Neuropathology, New York State Psychiatric Institute and Departments of Psychiatry, Columbia University, New York, NY, USA; 6 Department of Psychiatry and Biobehavioral Sciences, UCLA Semel Institute for Neuroscience, David Geffen School of Medicine, Los Angeles, CA, USA; 7 Department of Radiology, Henan Mental Hospital, the Second Affiliated Hospital of Xinxiang Medical University, Xinxiang, Henan, China; 8 Department of Psychiatry, Henan Mental Hospital, the Second Affiliated Hospital of Xinxiang Medical University, Xinxiang, Henan, China; 9 Henan Key Lab of Biological Psychiatry, Henan Mental Hospital, Xinxiang Medical University, Xinxiang, Henan, China

## Abstract

It is unclear whether abnormal spontaneous neural activation patterns found in chronic schizophrenia patients (CSP) are part of the pathogenesis of disease, consequences of chronic illness, or effects of antipsychotic treatment. We performed a longitudinal resting-state functional magnetic resonance imaging (fMRI) study in 42 treatment-naïve first-episode schizophrenia patients (FESP) at baseline and then after 8-weeks of risperidone monotherapy, and compared the findings to 38 healthy volunteers. Spontaneous brain activity was quantified using the fractional amplitude of low-frequency fluctuations (fALFF) and regional homogeneity (ReHo) and compared between patients and controls. Pretreatment, patients exhibited higher fALFF in left caudate compared with controls. After treatment, patients had elevated fALFF in bilateral putamen and right caudate, and increased ReHo in right caudate and left putamen. Greater increase of fALFF in the left putamen correlated with less improvement in positive symptoms. Thus, abnormalities of spontaneous neural activity in chronic schizophrenia is at least partly due to a medication effect. The observed post-treatment increase in striatal intrinsic activity may reflect counter-therapeutic functional adaptation to dopamine D2 receptor occupancy required for medication effects on psychosis.

Resting-state functional magnetic resonance imaging (rfMRI) studies have identified spontaneous brain function alterations in schizophrenia. Such rfMRI measurements, including amplitude of low frequency fluctuations (ALFF), fractional ALFF (fALFF) and regional homogeneity (ReHo), are indices of spontaneous brain activity. ReHo^1^ is used to represent the degree of synchronization of a given voxel with its nearest neighbors at a voxel wise level. While both ALFF^2^ and fALFF^3^ are utilized to evaluate the regional intensity of spontaneous fluctuations in blood oxygenation level dependent (BOLD) signal, relative to ALFF, fALFF can suppress non-specific signal components in rfMRI and is more sensitive and specific for detection of spontaneous brain activities. Cross-sectional studies have reported fALFF^4^ and ReHo^5^ alterations in the early phase of schizophrenia. More extensive fALFF^6^,^7^,^8^ and ReHo^9^,^10^ abnormalities have been observed in chronic schizophrenic patients (CSP), potentially an indication that the findings are the result of disease progression or effects of chronic illness. Alternatively the findings could be due to the effects of antipsychotic medication.

All known effective antipsychotic drugs are D2-4 dopamine receptor antagonists or partial agonists^11^, in cortical regions, such as prefrontal and cingulate cortices, and subcortical regions, such as basal ganglia (caudate, putamen and globus pallidus) and amygdala^12^. Dopamine (DA) modulates spontaneous brain activity. Levodopa regulates ALFF measures in main cortical output areas of the striatum and ReHo values in striatal-cortical loops in Parkinson’s disease (PD)^13^,^14^. Administration of amisulpride, an atypical antipsychotic, for seven days to healthy volunteers, alters fALFF receiving dopaminergic imput, with changed fALFF levels in putamen correlating with drug plasma concentration^15^. Finally, imaging genetics studies report associations between polymorphisms of DA-related genes and resting-state brain activities^16^,^17^,^18^. Collectively, these data imply a modulatory role of antipsychotic treatment in spontaneous brain activity. To date, one longitudinal study^19^ has examined the effect of 6-weeks of antipsychotic treatment on resting-state ALFF measures in 34 drug-naïve first-episode schizophrenia patients (FESP). Medications examined in that study included sulpiride, clozapine, olanzapine, risperidone and aripiprazole. Increased ALFF in frontal gyrus, parietal lobule, temporal gyrus and striatum was observed after treatment although the affected brain regions varied. The heterogeneity of drug binding affinities may potentially cloud the net effect on spontaneous brain activity. Positron emission tomography (PET) studies^20^,^21^ have shown that different classes of antipsychotics have different effects on neural metabolism and blood flow. To distinguish effects of medication from early disease effect requires a longitudinal study of drug-naïve FESP treated with standardized protocol-driven, antipsychotic monotherapy to separately determine disease effects and pharmacological effects on spontaneous brain activities.

Post-treatment alterations of fMRI measurements that correlate with symptom improvement may shed light on possible mechanisms of action of existing treatments. Previously published results, however, have varied in the nature of the associations between brain functional measurements and clinical symptoms, possibly due to differences in sample characteristics and medication heterogeneity. In 34 drug-naïve FESP treated with different antipsychotics, such as olanzapine, sulpiride, risperidone and clozapine, Lui *et al*.^19^ found that longitudinal increases of ALFF in frontal gyrus, parietal lobule, temporal gyrus and right caudate were negatively correlated with improvement of positive symptoms. In 23 antipsychotic-naïve FESP treated with amisulpride monotherapy, Nielsen *et al*.^22^ reported that the change of BOLD signals in ventral striatum showed positive correlation with the improvement of positive symptoms. In contrast, medication-naïve FESP treated with risperidone, olanzapine or quetiapine^23^ showed no correlation between improvement of symptoms and alteration of cortex activation examined by fMRI.

Therefore, we conducted a longitudinal fMRI study in a relatively large sample of drug-naïve FESP, within 12 months of psychosis onset, to quantify fALFF and ReHo both at baseline and after 8 weeks treatment with risperidone monotherapy. We also performed an exploratory analysis to explore whether alterations of ReHo or fALFF were related to changes in clinical symptoms. At baseline, we hypothesized that patients would exhibit abnormalities of fALFF and ReHo compared with healthy volunteers. We hypothesized that risperidone monotherapy would modulate ReHo and fALFF in regions with dopaminergic input, and that change would be related to clinical improvement.

## Results

### Demographic characteristics

The demographic characteristics of the participants are shown in Table 1. Healthy volunteers did not differ from patients with respect to age (p = 0.929), gender (p = 0.888), years of education (p = 0.373), and alcohol (p = 0.282) and tobacco use (p = 0.967).

### Longitudinal alterations of clinical symptoms

As shown in Table 1, after 8-week treatment, patients had pronounced reductions in Positive and Negative Syndrome Scale (PANSS) total symptom (PANSS-T), positive symptom (PANSS-P), and general psychopathology symptom (PANSS-G) scores, but no significant alterations in PANSS negative symptom scores (PANSS-N)

### Results of one-sample t-test within group

One-sample t-test showed higher standardized fALFF and ReHo values relative to whole brain in cortical regions, such as prefrontal and cingulate cortices, and subcortical regions, such as striatum (caudate, putamen and globus pallidus) in both patients group and controls group. It may reflect higher spontaneous neuronal activity in these brain areas during resting but awake state. (Supplementary Figures S1 and S2).

### Baseline alterations in fALFF and ReHo

**Figure 1:**
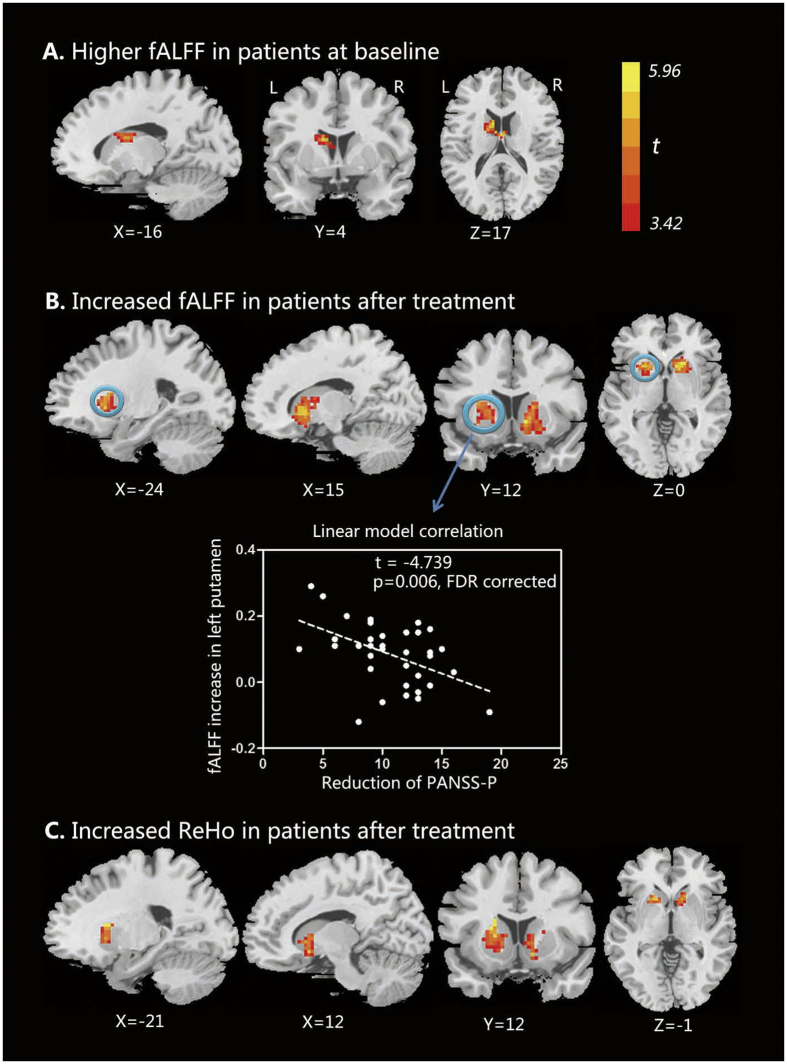
Comparison and correlation results of fALFF and ReHo. **(A)** At baseline, patients had higher fALFF in left caudate (p < 0.05, AlphaSim corrected) compared with healthy volunteers. **(B)** After 8-weeks of treatment, patients showed increased fALFF in right caudate and bilateral putamen (p < 0.05, AlphaSim corrected) relative to baseline. The associations between longitudinal increases of fALFF (follow-up minus baseline) and improvement of clinical symptoms (baseline PANSS scores minus follow-up PANSS scores) were evaluated by using multiple regression analysis with baseline PANSS scores, age and gender as control variables. Correlation analysis showed more posttreatment increases of fALFF in left putamen was associated with less improvement of positive symptoms (t = −4.739, p = 0.006, FDR corrected). **(C)** After treatment, patients showed elevated ReHo in right caudate and left putamen compared with baseline (p < 0.05, AlphaSim corrected). fALFF, fractional amplitude of low-frequency fluctuation; ReHo, regional homogeneity; PANSS, positive and negative syndrome scales; PANSS-P, PANSS positive symptom scores.

At baseline, patients had higher fALFF in left caudate (p < 0.05, AlphaSim corrected) compared with healthy volunteers (Fig. 1A, Table 2). Patients also had higher fALFF in right caudate and bilateral putamen, as well as ReHo in right caudate and left putamen (p < 0.005, uncorrected), although these differences did not survive multiple comparisons correction (Supplementary Table S1). No regions showed lower fALFF/ReHo in patients relative to healthy volunteers.

### Longitudinal alterations in fALFF and ReHo

After 8-weeks of treatment, fALFF increased in right caudate and bilateral putamen (p < 0.05, AlphaSim corrected) (Fig. 1B, Table 2), and ReHo increased in right caudate and left putamen compared with baseline (p < 0.05, AlphaSim corrected) (Fig. 1C, Table 2). No regions demonstrated decreased fALFF or ReHo after treatment.

### Relationships between longitudinal alterations of fMRI measures and clinical variables

Longitudinal increases of fALFF in left putamen were negatively correlated with improvement in PANSS-P scores (t = −4.739, p = 0.0006, FDR corrected) (Fig. 1B). The baseline fALFF of left putamen in patients showed trend-level positive correlation with the baseline PANSS-P scores (t = 2.297, p = 0.082, FDR corrected) (Supplementary Figure S3). There were no significant correlations between the longitudinal alterations of fALFF/ReHo and the changes in PANSS-T or PANSS-G symptom scores after treatment (Ps > 0.05).

## Discussion

To our knowledge, this is the first longitudinal study examining the effect of risperidone monotherapy on fALFF and ReHo in treatment-naïve FESP. Prior to treatment, patients had higher fALFF in left caudate, and a similar trend in the right caudate and bilateral putamen, as well as ReHo in right caudate and left putamen, relative to healthy volunteers. After 8 weeks of treatment, there was a further increase of fALFF in right caudate and bilateral putamen, and an increase in ReHo in right caudate and left putamen. As hypothesized, our results indicated that risperidone monotherapy altered ReHo and fALFF in brain regions within the dopaminergic pathway in FESP. Additionally, the degree of post-treatment increase of fALFF in the left putamen correlated with less improvement in positive symptoms. This study demonstrates that abnormalities of spontaneous neural activity documented in CSP may be partly a medication effect and partly due to the disease.

**Figure 2:**
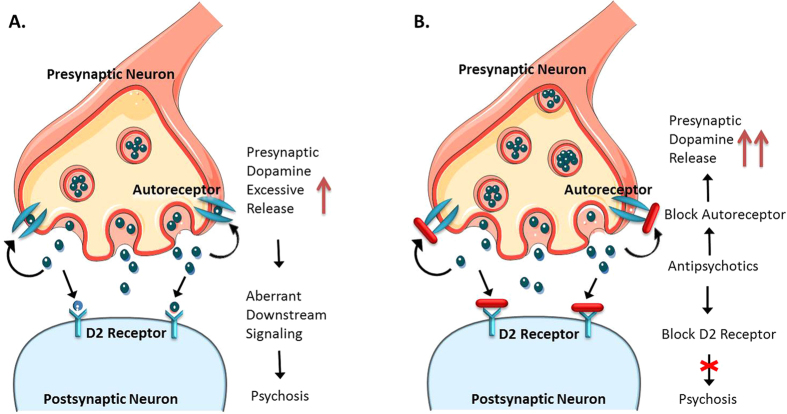
The proposed mechanism of higher striatal spontaneous neural activity at baseline and follow-up in FESP. (**A)** According to the DA hypothesis for schizophrenia^11^, the excessive release of striatal presynaptic DA is fundamental to the generation of the psychosis. Given the potential associations between DA and modulation of spontaneous cerebral function, we propose that the higher spontaneous neural activity of striatal region observed in this study may be underlied by the excessive release of striatal presynaptic DA. (**B)** Antipsychotic treatment ameliorates psychosis through blocking the striatal postsynaptic D2 dopamine receptors, which would compensatorily elevate striatal presynaptic dopamine (due to blockade presynaptic D2 autoreceptors) and further paradoxically deteriorate the pre-existing pathological abnormality, i.e. excessive release of striatal presynaptic dopamine. We propose that the posttreatment striatal hyperactivity observed in this study may result from treatment-induced compensatory increase of striatal presynaptic DA. FESP, treatment-naïve first-episode schizophrenia patients; DA, dopamine. Author M.L.H. drew the figure, and referenced a synapse template from Servier Medical Art (http://www.servier.hk/content/servier-medical-art).

Higher striatal rfMRI indices at baseline are consistent with elevated striatal neural activity in schizophrenia. Moreover, the higher spontaneous neural activity of striatal region (left putamen) correlated with greater severity of positive symptoms at a trend level, consistent with previous studies^4^,^11^,^24^,^25^ showing that the striatal overactivity is critically involved in the pathomechanism of schizophrenia, especially psychosis. According to the DA hypothesis for schizophrenia^11^, the excessive release of striatal presynaptic DA underlies psychosis (Fig. 2A). Given the potential associations between DA signals and spontaneous brain activity^13^,^14^,^16^, we propose that the higher spontaneous neural activity of striatal region observed in this study may be due to excessive release of striatal DA (Fig. 2A).

Post-treatment increases of fALFF in right caudate and bilateral putamen, as well as increases of ReHo in right caudate and left putamen, indicate that greater spontaneous neural activity in striatal areas after short-term risperidone monotherapy. Antipsychotic medications increase firing of DA neurons that project to striatal regions-including the putamen, caudate and palladium, and block D2-4 receptors, which both ameliorates positive symptoms and causes extrapyramidal symptoms^11^. Consequently, altered spontaneous neural activities in the caudate and putamen are not unexpected. Consistent with our findings, a longitudinal resting-state fMRI study^19^ reported increased striatal ALFF after 6 weeks of treatment in FESP. Moreover, PET and structural MRI studies have also demonstrated that antipsychotic cause activity increases^21^,^26^ and volume enlargement^27^,^28^ in striatum of schizophrenia patients. However, the post-treatment increases of striatal spontaneous activity observed in the present study may not be directly therapeutic. First, prior to treatment, patients have abnormally higher fALFF in left caudate, and a comparable trend in right caudate and bilateral putamen, as well as that of ReHo in right caudate and left putamen. Second, greater post-treatment increases of fALFF in left putamen was associated with less improvement in positive symptoms, and higher baseline fALFF in left putamen correlated with greater severity of positive symptoms prior to treatment on a trend level. Furthermore, the DA hypothesis for schizophrenia proposes that excessive release of ventral or limbic striatal DA reflects part of the pathogenesis of psychosis, and antipsychotic treatment ameliorates psychosis through blocking striatal postsynaptic D2-4 DA receptors. That action, in turn leads to elevated striatal DA release (due to blockade presynaptic D2 autoreceptors) and potentially increased D1/5 signaling since D2-4 receptors are blocked by the medication^11^. Given the previous evidence suggesting potential associations between DA and modulation of spontaneous brain activity^13^,^17^, we propose that the posttreatment striatal hyperactivity observed in our current study may result from treatment-induced compensatory increase of ventral striatal DA release (Fig. 2B). In addition, risperidone is also a strong 5-HT_2A_ antagonist, but there is little evidence suggesting potential associations between 5-HT and modulation of spontaneous brain activity. The increase in the magnitude of the spontaneous activity in the striatum cannot be readily explained by an effect of 5-HT_2A_ antagonism. Overall, posttreatment increases of striatal spontaneous activities observed in this study may be treatment-induced hyperactivity, which could reflect ineffective functional adaptation to D2-4 receptor occupancy and may mitigate the treatment response of psychosis. It is noteworthy that antipsychotics acutely cause increases in DA release in response to receptor blockade and an increase in presynaptic DA synthesis, in contrast, subacute medications can decrease it^11^,^29^,^30^. Future longer-term follow-up studies in FESP are needed to clarify how changes in striatal intrinsic function progresses over time and is linked to changes in DA release and signaling.

We found alterations of ReHo/fALFF after risperidone monotherapy treatment in striatum but not extrastriatal regions, in contrast to the findings of Lui *et al*.^19^ that found both striatum and extrastriatal regions, such as frontal and temporal cortices demonstrated increases of ALFF in FESP after short-term treatment. One potential explanation for the differences between the findings of these studies may be the difference in medications administered, as in their study patients were treated with an array of drugs, including quetiapine, clozapine, olanzapine, sulpiride, aripiprazole and risperidone. Different antipsychotic agents produce different patterns of occupancy. For example, clozapine and quetiapine produce preferentially block DA D2 receptors in temporal cortex compared with putamen^31^; olanzapine and risperidone have less affinity for extrastriatal DA D2 receptors^32^,^33^; while aripiprazole shows comparable DA D2 receptor blockade in striatal and extrastriatal areas^34^. Although the work of Lui *et al*. also demonstrated ALFF alterations of both striatal and extrastriatal regions in a subgroup of patients treated with risperidone monotherapy, the relatively small sample (n = 12) in the subgroup may explain the differences between their results and our current findings, which were confined to striatum.

Although measurements of spontaneous neural activity appear to have moderate test-retest reliability^35^, the lack of repeated fMRI scans for the healthy volunteer group is a limitation of our present study as we cannot fully exclude the possibility that the posttreatment changes of ReHo/fALFF might be related to a time-dependent change over the test-retest period. However, we did formally rule out the impact of simply having repeated fMRI scans. Although ReHo and fALFF are considered to represent spontaneous brain activity, there remains a lack of consensus regarding the exact physiological basis of the two measures, which will need to be further examined in future studies. Given that a recent paper highlighted the clusterwise interference is problematic, we added a discussion about the method (See supplementary discussion). Finally, future studies exploring different antipsychotics effects in several clinical subgroups with large samples of treatment-naïve FESP are needed to further clarify the potential heterogeneous effects of antipsychotic agents on intrinsic neural activity.

In conclusion, this study found that short-term risperidone monotherapy results in increased striatal intrinsic activity in drug-naïve FESP, overlapping with brain regions that exhibit abnormalities of spontaneous neural activity prior to treatment. Posttreatment increases of striatal intrinsic activities may represent ineffective functional adaptation to D2 receptor blockade and moderate the treatment effects on psychosis. These results provide new insight into antipsychotic medication effects on resting-state neural function, and offer promise that fALFF and ReHo measures can be used as potential biomarkers for tracking long-term medication effects to individualize patient care. In the future, therefore, longer-term follow-up pharmacological studies in FESP may clarify how changes in intrinsic neural function progress over time and how these are associated with the occurrence of medication resistance in some individuals.

## Methods

### Participants

Forty-two treatment-naïve FESP and 38 age-, gender-, handedness-, and education-matched healthy volunteers were recruited from the Second Affiliated Hospital, Xinxiang Medical University, China (Table 1). All the participants were also included in our previously published longitudinal study of MR spectroscopy data^36^. In that previous study, we collected ^1^H proton magnetic resonance spectroscopy data to explore the N-acetylaspartate level, while in the current study we collected rfMRI data to explore the spontaneous brain activity, and the rfMRI data presented here have not been previously published. Patients were diagnosed by at least two trained psychiatrists using the Structured Clinical Interview for DSM-IV-TR, patient version(SCID-I/P). They were suffering from their first episode of schizophrenia and were free of other DSM-IV diagnoses or clinically significant medical disease. They had less than 1 year of illness duration. Healthy volunteers were screened according to the SCID-NP(Non-Patient Edition) for lifetime absence of any psychiatric disorder.

This study was carried out in accordance with the Declaration of Helsinki. The Ethics Committee of the Second Xiangya Hospital of Central South University (No. S088, 2012) and the cooperated hospital, the Second Affiliated Hospital of Xinxiang Medical University approved this study, and written informed consent from all subjects were obtained. All participants were permitted to withdraw from the study at any time. All patients had MRI scans within 24 hours after enrollment in this study, a time when they were free from drug. Post-treatment scans were scheduled for patients after an 8-week treatment course. One patient’s fMRI data at baseline was excluded due to quality control, and 4 patients withdrew from the follow-up MRI scans. We collected fMRI data from 41 FESP at baseline, 38 patients after treatment and 38 healthy volunteers.

### Therapy and Clinical Assessments

All patients were stabilized on risperidone monotherapy at a dosage of 4–6 mg/day for 8 weeks. Mood stabilizers and antidepressants were not used. The efficacy and safety of risperidone was assessed weekly by clinical interviews. During the treatment, no serious adverse effects occurred. Symptoms severity of all patients (n = 42) were evaluated at baseline and follow-up with the 30-item Positive and Negative Syndrome Scale (PANSS)^37^.

### fMRI imaging

Imaging data were collected using a 3T magnetic resonance imager (Siemens Verio, Erlangen, Germany). Participants were fitted with earplugs and foam pads to reduce scanner noise and limit head motion. Participants were instructed to keep their eyes closed, relax, but not fall asleep during scanning. RfMRI was completed using a gradient-echo echo-planar imaging (GE-EPI) with following parameters: TR/TE = 2000/30 ms, FOV = 240 × 240 mm^2^, flip angle = 90°, matrix size = 64 × 64, 240 volumes, slice thickness = 3 mm, slice gap = 0, and voxel size = 3.75 × 3.75 × 3 mm^3^. High resolution sagittal T1-weighted anatomical scans were obtained with a spoiled gradient echo (SPGR) pulse sequence (TR/TE = 1900/2.52ms, FOV = 250 × 250 mm^2^, flip angle = 9°, slice thickness = 1 mm, slice gap = 0, 176 slices). Patients were scanned both at baseline and follow-up, while healthy volunteers underwent once.

### Image preprocessing

The Data Processing Assistant for Resting-State fMRI (DPARSF)^38^ which is based on Statistical Parametric Mapping (SPM, http://www.fil.ion.ucl.ac.uk/spm) and the toolbox for Data Processing & Analysis of Brain Imaging (DPABI, http://rfmri.org/DPABI)^39^ were used to preprocess the fMRI image. To avoid non-equilibrium effects of magnetization, we discarded the first 10 volumes for each participant. Slice timing and realignment correction were performed. Any subject with head motion >1.5 mm translation or >1.5° rotation in any direction was removed. The data were then normalized to standard EPI template in SPM (resampled voxel size 3 × 3 × 3 mm^3^). We also performed another T1 co-registration analysis to process the data (The detailed methods and results showed in supplementary information).

### Calculation of fALFF

The resampled images were smoothed with a Gaussian kernel of 8 mm and detrended to remove the linear signal drift. Next, nuisance variables (including Friston 24-parameter model head motion, white matter (WM) signal and cerebral spinal fluid (CSF) signal) were regressed from the data. The fALFF of all subjects were calculated based on the method of Zou *et al*.^3^. Finally, the raw fALFF value of each voxel was divided by the global average fALFF value for standardization.

### Calculation of ReHo

The resampled images were detrended and regressed the Friston 24 head motion, WM and CSF signal. Then the band-pass filtering (0.01–0.08 Hz) was performed to remove the effects of high-frequency noise. ReHo maps^1^ were conducted by calculating Kendall’s coefficient of concordance for a given voxel time series with those of its nearest 26 neighbors. For standardization purpose, the ReHo value of each voxel was divided by the whole brain mean ReHo value.

### Group-level Analysis of fALFF and ReHo

The DPABI toolbox was used to conduct group-level analyses of fALFF and ReHo. One-sample t-test was performed to indicate the baseline maps of fALFF and ReHo. Two-sample t-test was used to analyze group differences between patients at baseline and healthy volunteers, with age and gender as control covariates. Paired-sample t-test was used to assess longitudinal changes of the rfMRI measures In patients at baseline and follow-up. Multiple correction was performed using cluster-extent correction (AlphaSim) as implemented through DPABI, and the parameters were set as follows: individual voxel threshold p = 0.001, Number of Monte Carlo simulations = 1000, and p = 0.05 as the effective threshold for cluster-extent correction.

We performed a whole brain approach by computing fALFF and ReHo value for every brain voxel and then doing group level analysis. Based on the literature^11^,^12^,^15^,^19^,^22^, we hypothesized that risperidone monotherapy would modulate ReHo and fALFF of regions within dopaminergic pathway in patients. Even though we had such brain region specific hypotheses, a brain-wide voxel approach can both test whether these regions show the abnormality or treatment effect, and demonstrate that neighboring regions do not show the effect. That approach both supports the hypothesized brain regions as being involved and excludes the non-hypothesized brain regions.

The brain regions with significant longitudinal changes of rfMRI measures were individually extracted as masks, and then the masks were used to extract the mean fALFF and ReHo values respectively from patients at baseline and follow-up. The associations between baseline fALFF or ReHo and baseline symptoms were evaluated by using multiple regression analysis with age and gender as control variables. The associations between longitudinal alterations of fALFF or ReHo (follow-up minus baseline) and improvement of clinical symptoms (baseline PANSS scores minus follow-up PANSS scores) were evaluated by using multiple regression analysis with baseline PANSS scores, age and gender as control variables. We performed False Discovery Rate (FDR)^40^ correction in n_1_ (number of regions showing significant posttreatment alterations of ReHo or fALFF) ×n_2_ (numbers of PANSS dimensions showing significant longitudinal alterations) multiple tests (cut-off value 0.05) for the correlation analysis.

### Statistical Analysis

Two-sample T-tests were used to compute the differences in age and years of education between FESP and healthy volunteers; and chi-square tests were performed to assess the differences in gender, alcohol and tobacco use between the two groups. Paired-sample T-test was used to compare patients’ longitudinal alterations of PANSS scores. Threshold of statistical significance was p < 0.05.

## Additional Information

**How to cite this article**: Hu, M.-L. *et al*. Short-term Effects of Risperidone Monotherapy on Spontaneous Brain Activity in First-episode Treatment-naïve Schizophrenia Patients: A Longitudinal fMRI Study. *Sci. Rep.* **6**, 34287; doi: 10.1038/srep34287 (2016).

## Supplementary Material

Supplementary Information

## Figures and Tables

**Table 1:** Demographic and clinical characteristics for first-episode schizophrenia patients both at baseline and follow-up and their matched healthy controls.

Variable	Patients at Baseline (n = 42)	Patients at Follow-up (n = 42)	Controls (n = 38)	Analysis^a^		Analysis^b^	
	M ± SD	M ± SD	M ± SD	t	P	t	P
Age(years)	24.86 ± 4.80		24.76 ± 4.56	0.09	0.929		
Education(years)	10.48 ± 2.84		11.05 ± 2.91	0.90	0.373		
Duration of illness(month)	8.38 ± 2.61						
PANSS-T	91.90 ± 11.23	67.24 ± 10.10				13.19	<0.001
PANSS-P	25.60 ± 3.75	15.83 ± 3.28				14.58	<0.001
PANSS-N	18.17 ± 5.21	17.07 ± 4.86				1.42	0.163
PANSS-G	48.14 ± 6.46	34.33 ± 4.71				13.37	<0.001
	n		n	*χ*^2^	P	*χ*^2^	P
Gender(male/female)	27/15		25/13	0.02	0.888		
Alcohol use	6		9	1.16	0.282		
Tobacco use	9		8	0.002	0.967		
Family history of psychiatric illness	15		0				
Handedness (right)	42		38				

^a^Patients at baseline *vs.* controls, independent-samples T-test.

^b^Patients at baseline *vs.* follow-up, paired-samples T-test.

One patient’s baseline fMRI data was excluded due to the poor quality, and 4 patients withdrew from the follow-up MRI scans. Finally, we collected fMRI data from 41 FESP at baseline, 38 patients after treatment and 38 healthy volunteers. Symptoms severity of all patients (n = 42) was evaluated both at baseline and follow-up with the 30-item Positive and Negative Syndrome Scale (PANSS). PANSS-T = PANSS total scores; PANSS-P = PANSS positive symptom scores; PANSS-N = PANSS negative symptom scores; PANSS-G = PANSS general psychopathological symptom scores; FESP, first-episode schizophrenia patients.

**Table 2:** Baseline and longitudinal alterations in fALFF and ReHo (p < 0.05, AlphaSim corrected).

Brain region	AAL	MNI coordinates			Voxels	Maximat *t* value
		X	Y	Z		
Patient at baseline *vs* Controls						
fALFF, left caudate	71	−15	3	18	52	5.96
Patient at follow-up *vs* Baseline						
fALFF, right caudate	72	12	15	0	71	5.43
fALFF, left putamen	73	−21	15	3	38	5.24
fALFF, right putamen	74	21	18	0	50	5.39
ReHo, right caudate	72	12	12	−9	20	4.75
ReHo, left putamen	73	−21	12	12	20	5.12

AAL = Automated Anatomical Labeling; MNI = Montreal Neurological Institute; fALFF = fractional amplitude of low-frequency fluctuation; ReHo = regional homogeneity.

Multiple correction was performed using cluster-extent correction (AlphaSim) as follows: individual voxel threshold p = 0.001, Number of Monte Carlo simulations = 1000, and p = 0.05 as the effective threshold for cluster-extent correction.
