# Supplementary material for: Short-term Effects of Risperidone Monotherapy on Spontaneous Brain Activity in First-episode Treatment-naïve Schizophrenia Patients: A Longitudinal fMRI Study
Source: Sci Rep. 2016 Oct 4;6:34287. doi: 10.1038/srep34287 (PMC5048418; doi:10.1038/srep34287)
Supplement: Supplementary Information [file srep34287-s1.doc]

Supplementary Information for

Short-term Effects of Risperidone Monotherapy on Spontaneous Brain Activity in First-episode Treatment-naïve Schizophrenia Patients: A Longitudinal fMRI Study

Mao-Lin Hu^1,2,9*^, Xiao-Fen Zong^1,2,9*^, Jun-Jie Zheng^3^, Spiro P. Pantazatos^4^, Jeffrey M. Miller^2^, Zong-Chang Li^1^, Yan-Hui Liao^1,5^, Ying He^1^, Jun Zhou^1^, De-En Sang^6^, Hong-Zeng Zhao^6^, Lu-Xian Lv^7,8^, Jin-Song Tang^1,5^, J. John Mann^2^ & Xiao-Gang Chen^1,9^

^1^ Department of Psychiatry, the Second Xiangya Hospital, Central South University, Changsha, Hunan, China.

^2^ Division of Molecular Imaging and Neuropathology, New York State Psychiatric Institute and Departments of Psychiatry and Radiology, Columbia University, New York, NY, USA.

^3^ Key Laboratory for NeuroInformation of the Ministry of Education, School of Life Science and Technology, University of Electronic Science and Technology of China, Chengdu, China.

^4^ Division of Molecular Imaging and Neuropathology, New York State Psychiatric Institute and Departments of Psychiatry, Columbia University, New York, NY, USA.

^5^ Department of Psychiatry and Biobehavioral Sciences, UCLA Semel Institute for Neuroscience, David Geffen School of Medicine, Los Angeles, CA , USA.

^6^ Department of Radiology, Henan Mental Hospital, the Second Affiliated Hospital of Xinxiang Medical University, Xinxiang, Henan, China.

^7^ Department of Psychiatry, Henan Mental Hospital, the Second Affiliated Hospital of Xinxiang Medical University, Xinxiang, Henan, China.

^8^ Henan Key Lab of Biological Psychiatry, Henan Mental Hospital, Xinxiang Medical University, Xinxiang, Henan, China.

^9^ Mental Health Institute of the Second Xiangya Hospital, Central South University, Chinese National Clinical Research Center on Mental Disorders (xiangya), Chinese National Technology Institute on Mental Disorders, Hunan Key Laboratory of Psychiatry and Mental Health, Changsha, Hunan, China.

Correspondence and requests for materials should be addressed to X.G.C. (chenxghn@gmail.com); J.S.T. (tangjinsonghn@gmail.com) or J.J.M. ( jjm@columbia.edu).

Corresponding Address: Mental Health Institute of the Second Xiangya Hospital, Central South University, 139 Middle Renmin Road, Changsha, Hunan 410011, China.

* These authors contributed equally to this work.

*
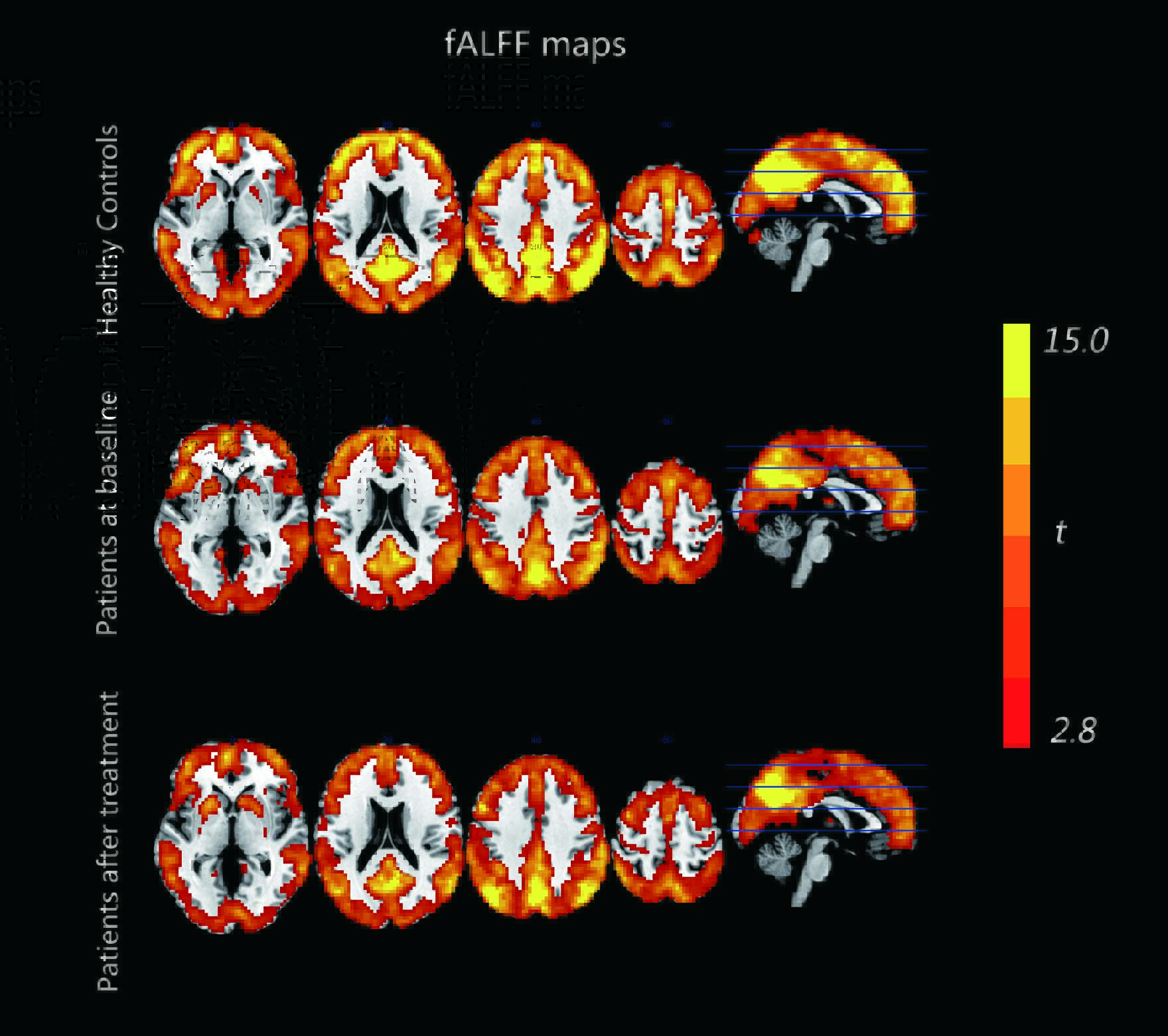
*

Figure S1. Results of the one-sample t-test within group. The fALFF maps showed significant higher standardized fALFF value in the cortical regions, such as frontal and cingulate cortices, and subcortical regions, such as striatum (caudate, putamen and globus pallidus).

*
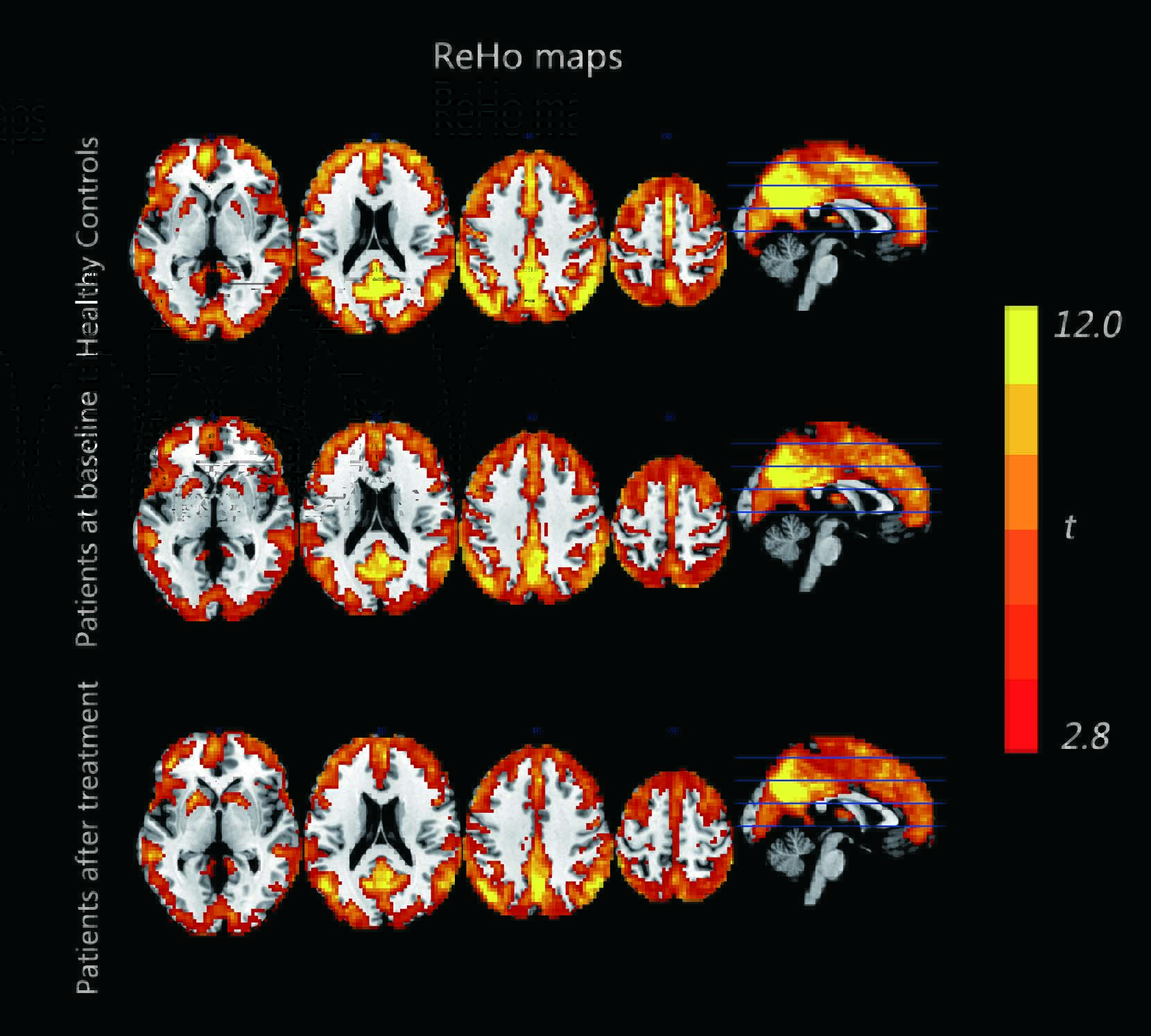
*

Figure S2. Results of the one-sample t-test within group. ReHo maps showed significant higher standardized ReHo value in the cortical regions, such as frontal and cingulate cortices, and subcortical regions, such as striatum (caudate, putamen and globus pallidus).

**Table S1. Increased fALFF and ReHo in patients *vs* controls at baseline (p<0.005, uncorrected)**

| **Brain region** | **AAL** | **MNI coordinates** | | | **Voxels** | **Maximat *t* value** |
| --- | --- | --- | --- | --- | --- | --- |
|  |  | **x** | **y** | **z** |  |  |
| fALFF | | | | | | |
| Right caudate | 72 | 6 | 12 | -3 | 22 | 4.646 |
| Left putamen | 73 | -15 | 7 | -5 | 15 | 3.544 |
| Right putamen | 74 | 17 | 9 | -9 | 4 | 3.187 |
| ReHo | | | | | | |
| Right caudate | 72 | 18 | 3 | 21 | 19 | 5.271 |
| Left putamen | 73 | -21 | 3 | 12 | 5 | 3.242 |

fALFF= fractional amplitude of low-frequency fluctuation; ReHo= regional homogeneity; AAL= Automated Anatomical Labeling; MNI= Montreal Neurological Institute.


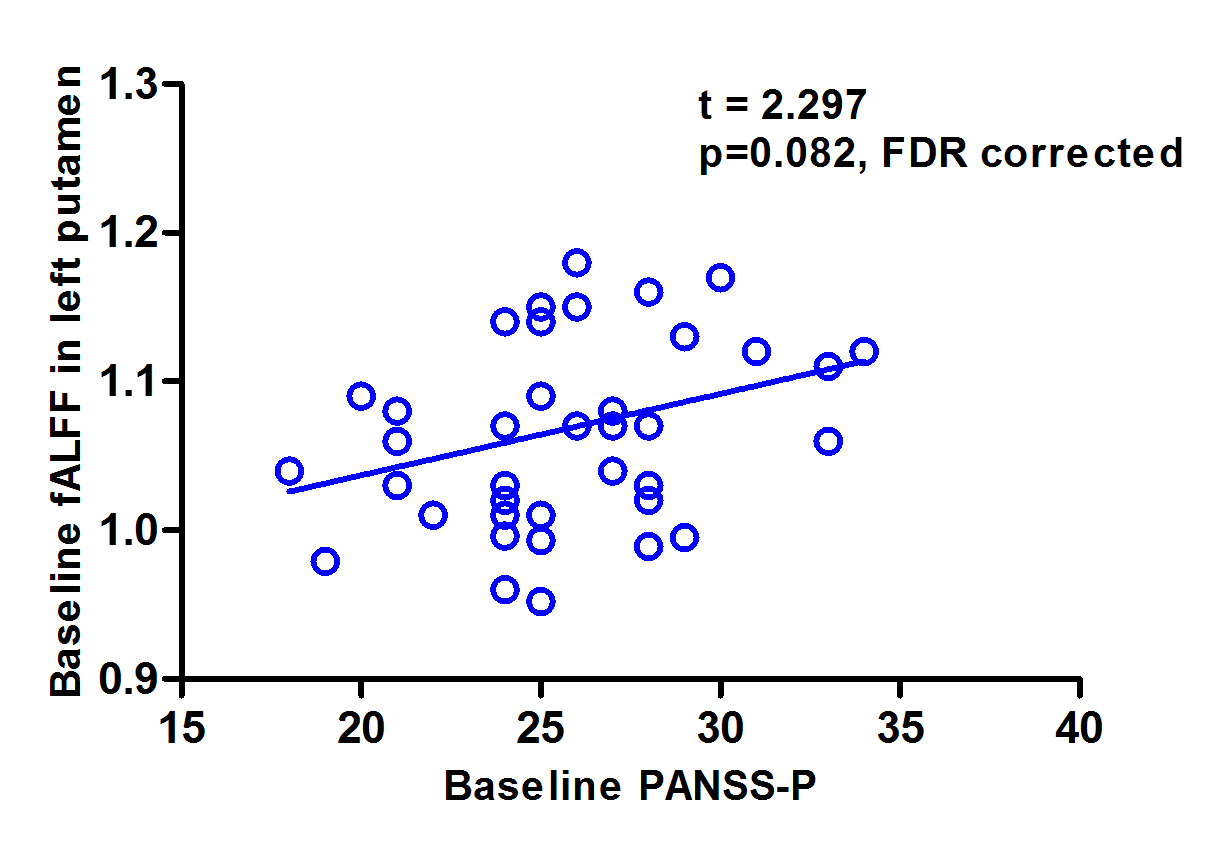


**Figure S3.** The association between fALFF of left putamen and positive symptoms in patients at baseline were evaluated by using multiple regression analysis with age and gender as control variables. The baseline fALFF of left putamen in patients showed trend-level positive correlation with the baseline PANSS-P scores (t=2.297, p=0.082, FDR corrected). fALFF, fractional amplitude of low-frequency fluctuation; FDR, false discovery rate; PANSS, positive and negative syndrome scales; PANSS-P, PANSS positive symptom scores.

**Supplementary analysis using T1 co-registration**

We also collected high-resolution T1-weighted images with a spoiled gradient echo (SPGR) pulse sequence (TR/TE=1900/2.52ms, FOV=250×250 mm^2^, flip angle=9°, slice thickness=1 mm, slice gap=0, 176 slices).

We performed an analysis with T1 co-registration. (1) Discarding the first 10 volumes for each participant; (2) Slice Timing; (3) Realignment; (4) Coregistering T1 images to fMRI images; (5) Structural images were segmented into gray matter (GM), white matter (WM), and cerebrospinal fluid (CSF) using the new segment option in SPM12 (http://www.fil.ion.ucl.ac.uk/spm); and then a common space reference template, representative of both groups was created using Diffeomorphic Anatomical Registration Through Exponentiated Lie algebra (DARTEL) [^1^](#_ENREF_1); (6) Normalizing by using T1 image DARTEL segmented template.

**Calculation of fALFF:** Smooth by DARTEL (6×6×6mm); Remove linear detrend; Nuisance variables (including Friston 24-parameter model head motion, WM signal and CSF signal) were regressed from the data. The fALFF of all subjects was calculated based on the method of Zou et al [^2^](#_ENREF_2). Finally, the raw fALFF value of each voxel was divided by the global average fALFF value for standardization.

**Calculation of ReHo:** Remove linear detrend from DARTEL normalized images; Regressed the Friston 24 head motion, WM and CSF signal; Band-pass filter (0.01 Hz-0.08 Hz); ReHo maps [^3^](#_ENREF_3) were conducted by calculating Kendall’s coefficient of concordance for a given voxel time series with those of its nearest 26 neighbors. For standardization purpose, the ReHo value of each voxel was divided by the whole brain mean ReHo value.

**Results:** Group-level analysis and correlation analysis are the same as the methods described in the manuscript. This methodological approach yielded similar results as our previous analysis. At baseline, patients had higher fALFF in left caudate (p=0.043, AlphaSim corrected) compared with healthy volunteers (Supplementary Table S2, Figure S4A). After 8-weeks of treatment, patients showed increased fALFF in right caudate (p=0.002, AlphaSim corrected) and a similar trend in left putamen (p=0.075, AlphaSim corrected) (Supplementary Table S2, Figure S4B). After treatment, patients showed elevated ReHo in right caudate (p=0.003, AlphaSim corrected) and a similar trend in left putamen (p=0.088, AlphaSim corrected) compared with baseline (Supplementary Table S2, Figure S4C).

The baseline fALFF of left putamen in patients showed positive correlation with the baseline PANSS-P scores (t=2.128, p=0.042, FDR corrected) (Supplementary Figure S5A). Longitudinal increases of fALFF in left putamen were negatively correlated with improvement in PANSS-P scores (t= -2.36, p=0.032) (Supplementary Figure S5B).

Table S2. Baseline and longitudinal alterations in fALFF and ReHo (T1 co-registration method)

| Brain region | AAL | MNI coordinates | | | Voxels | Maximat  *t* value | *P* value (AlphaSim corrected) |
| --- | --- | --- | --- | --- | --- | --- | --- |
|  |  | X | Y | Z |  |  |  |
| Patient at baseline *vs* Controls | | | | | | |  |
| fALFF, left caudate | 71 | -12 | 3 | 18 | 28 | 4.84 | 0.043 |
| Patient at follow-up *vs* Baseline | | | | | | |  |
| fALFF, right caudate | 72 | 18 | 18 | 9 | 33 | 5.64 | 0.002 |
| fALFF, left putamen | 73 | -21 | 15 | 3 | 10 | 4.14 | 0.075 |
| ReHo, right caudate | 72 | 17 | 18 | 8 | 33 | 5.64 | 0.003 |
| ReHo, left putamen | 73 | -18 | 13 | 0 | 10 | 3.58 | 0.088 |

AAL= Automated Anatomical Labeling; MNI= Montreal Neurological Institute; fALFF= fractional amplitude of low-frequency fluctuation; ReHo= regional homogeneity. Multiple correction was performed using cluster-extent correction (AlphaSim) as follows: individual voxel threshold p=0.001, Number of Monte Carlo simulations=1000, and α=0.05 as the effective threshold for cluster-extent correction.

*
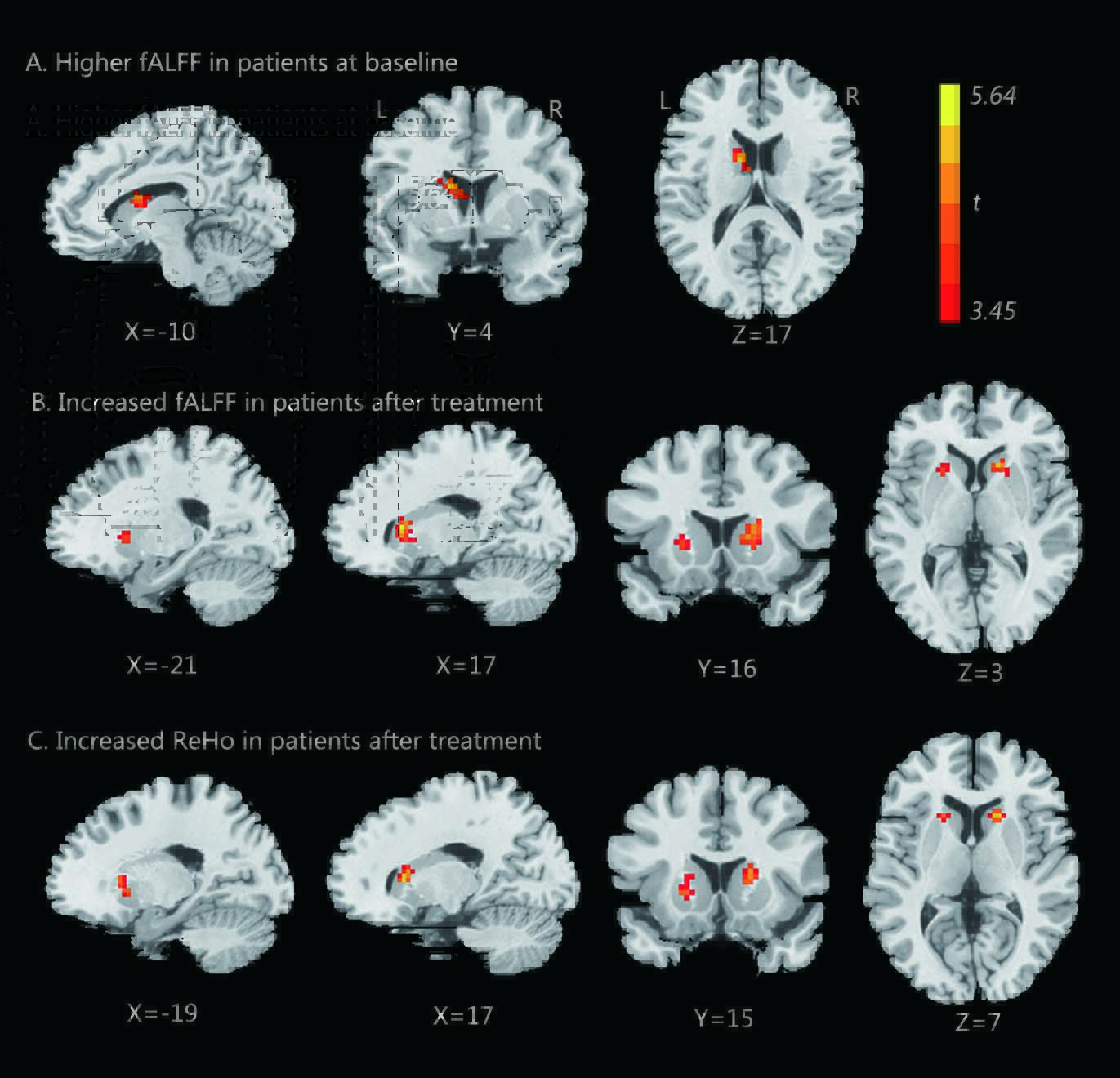
*

Figure S4 . Comparison results of fALFF and ReHo. (A) At baseline, patients had higher fALFF in left caudate (p=0.043, AlphaSim corrected) compared with healthy volunteers. (B) After 8-weeks of treatment, patients showed increased fALFF in right caudate (p=0.002, AlphaSim corrected) relative to baseline. The fALFF in left putamen also showed a similar trend (p=0.075, AlphaSim corrected). (C) After treatment, patients showed elevated ReHo in right caudate (p=0.003, AlphaSim corrected) and a similar trend in left putamen (p=0.088, AlphaSim corrected) compared with baseline. fALFF, fractional amplitude of low-frequency fluctuation; ReHo, regional homogeneity.

*
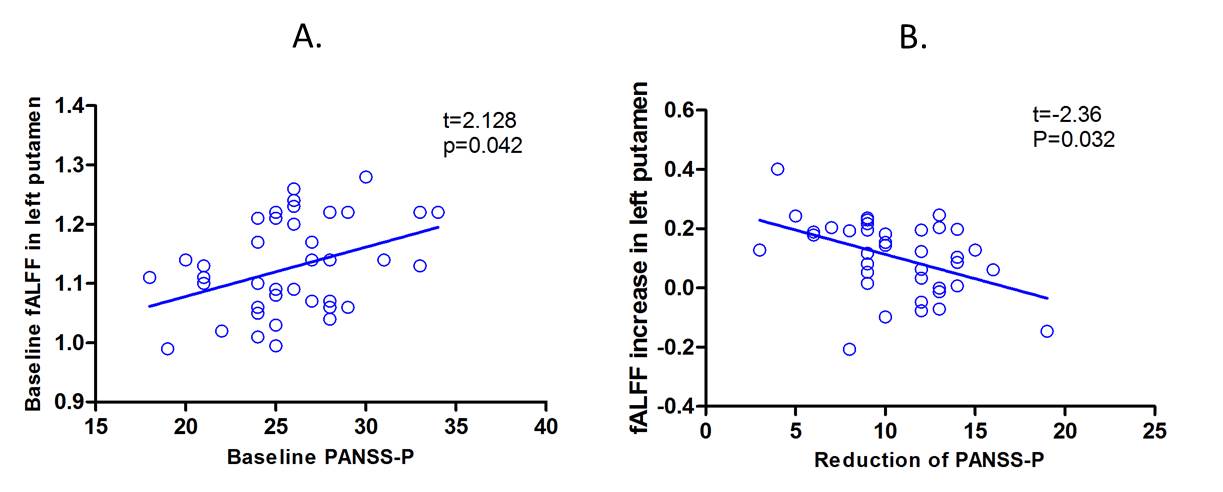
*

Figure S5. Relationships between fMRI measures and clinical variables. (A) The baseline fALFF of left putamen in patients showed positive correlation with the baseline PANSS-P scores (t=2.128, p=0.042). (B) Correlation analysis showed more posttreatment increases of fALFF in left putamen was associated with less improvement of positive symptoms (t=-2.36, p=0.032). fMRI, functional magnetic resonance imaging; fALFF, fractional amplitude of low-frequency fluctuation; PANSS, positive and negative syndrome scales; PANSS-P, PANSS positive symptom scores.

**Supplementary discussion**

Eklund *et al.* [^4^](#_ENREF_4) found most cluster-based parametric methods implemented in standard processing packages (i.e. SPM, FSL and AFNI) had inflated familywise Type I error rates, and that this inflation was worse for lower cluster-defining thresholds (i.e. p=0.01). Concerns were also raised about simulation-based methods such as AFNI’s 3dClustSim tool: 1) a 15 year old bug in 3dClustSim led to systematically lower p-values and 2) group smoothness as estimated by AFNI may be too low. Cluster-based inference in DPABI uses Matlab routines which apply Monte Carlo simulations similar to 3dClustSim ( <https://github.com/Chaogan-Yan/DPABI/blob/master/StatisticalAnalysis/y_AlphaSim.m> ). The DPABI V2.1 AlphaSim is unaffected by the bug mentioned in Eklund et. al. In addition, we set the cluster-determining voxel significance threshold at a recommended and more stringent P<0.001. Finally, DPABI uses a different approach to estimate group smoothness than AFNI (i.e. it is estimated from the 4D residuals, see http://rfmri.org/content/intrinsic-smoothness-estimation.

References

1 Ashburner, J. A fast diffeomorphic image registration algorithm. *NeuroImage* **38**, 95-113, doi:10.1016/j.neuroimage.2007.07.007 (2007).

2 Zou, Q. H. *et al.* An improved approach to detection of amplitude of low-frequency fluctuation (ALFF) for resting-state fMRI: fractional ALFF. *Journal of neuroscience methods* **172**, 137-141, doi:10.1016/j.jneumeth.2008.04.012 (2008).

3 Zang, Y., Jiang, T., Lu, Y., He, Y. & Tian, L. Regional homogeneity approach to fMRI data analysis. *NeuroImage* **22**, 394-400, doi:10.1016/j.neuroimage.2003.12.030 (2004).

4 Eklund, A., Nichols, T. E. & Knutsson, H. Cluster failure: Why fMRI inferences for spatial extent have inflated false-positive rates. *Proceedings of the National Academy of Sciences of the United States of America* **113**, 7900-7905, doi:10.1073/pnas.1602413113 (2016).
